# Larval habitat stability and productivity in two sites in Southern Ghana

**DOI:** 10.1186/s12936-023-04498-2

**Published:** 2023-03-02

**Authors:** Akua O. Forson, Isaac A. Hinne, Isaac Kwame Sraku, Yaw A. Afrane

**Affiliations:** 1 Department of Medical Laboratory Science, School of Biomedical and Allied Health Sciences, University of Ghana, Korle-Bu, Accra, Ghana; 2 Department of Medical Microbiology, University of Ghana Medical School, University of Ghana, Korle-Bu, Accra, Ghana

**Keywords:** *Anopheles gambiae* sensu lato, Larval habitat productivity, Larval habitat stability, Physicochemical parameters, Ghana

## Abstract

**Background:**

Mosquito larval source management (LSM) is a valuable additional tool for malaria vector control. Understanding the characteristics of mosquito larval habitats and its ecology in different land use types can give valuable insight for an effective larval control strategy. This study determined the stability and productivity of potential anopheline larval habitats in two different ecological sites: Anyakpor and Dodowa in southern Ghana.

**Methods:**

A total of 59 aquatic habitats positive for anopheline larvae were identified, and sampled every two weeks for a period of 30 weeks using a standard dipping method. Larvae were collected using standard dippers and were raised in the insectary for identification. Sibling species of the *Anopheles gambiae *sensu lato (s.l.) were further identified by polymerase chain reaction. The presence of larval habitats, their stability and larvae positive habitats were compared between the two sites using Mann–Whitney U and the Kruskal–Wallis test. Factors affecting the presence of *An. gambiae* larvae and physicochemical properties at the sites were determined using multiple logistic regression analysis and Spearman’s correlation.

**Results:**

Out of a total of 13,681 mosquito immatures collected, 22.6% (3095) were anophelines and 77.38% (10,586) were culicines*.* Out of the 3095 anophelines collected, *An. gambiae *s.l. was predominant (99.48%, n = 3079), followed by *Anopheles rufipes* (0.45%, n = 14), and *Anopheles pharoensis* (0.064%, n = 2). Sibling species of the *An. gambiae* consisted of *Anopheles coluzzii* (71%), followed by *An. gambiae *s.s. (23%), and *Anopheles melas* (6%). *Anopheles* mean larval density was highest in wells [6.44 (95% CI 5.0–8.31) larvae/dip], lowest in furrows [4.18 (95% CI 2.75–6.36) larvae/dip] and man-made ponds [1.20 (95% CI 0.671–2.131) larvae/dip].The results also revealed habitat stability was highly dependent on rainfall intensity, and *Anopheles* larval densities were also dependent on elevated levels of pH, conductivity and TDS.

**Conclusion:**

The presence of larvae in the habitats was dependent on rainfall intensity and proximity to human settlements. To optimize the vector control measures of malaria interventions in southern Ghana, larval control should be focused on larval habitats that are fed by underground water, as these are more productive habitats.

**Graphical Abstract:**

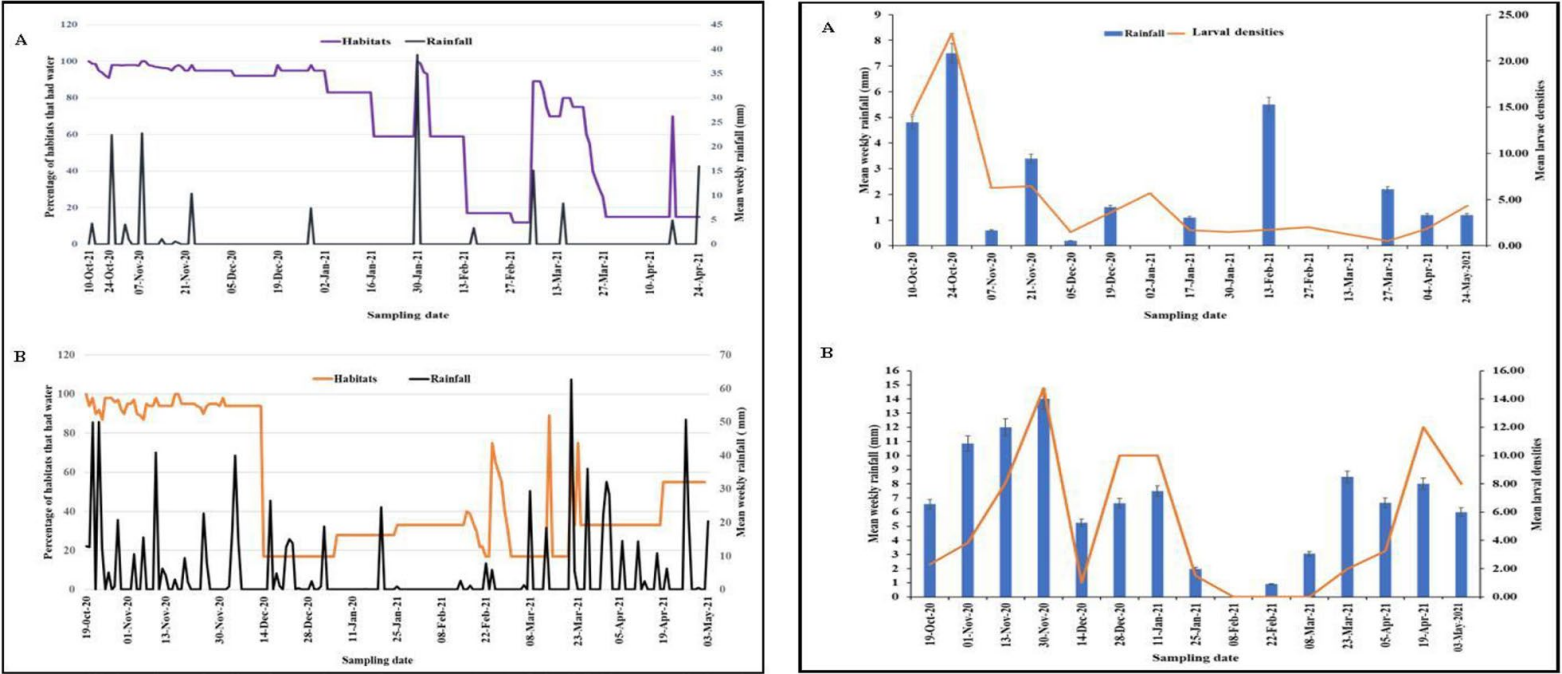

**Supplementary Information:**

The online version contains supplementary material available at 10.1186/s12936-023-04498-2.

## Background

*Anopheles* mosquitoes are an important vector of malaria and lymphatic filariasis in sub-Saharan Africa [1, 2]. The distribution of long-lasting insecticide-treated nets (LLINs) and indoor residual spraying (IRS) has been profound in aiding the reduction of a malaria vector population [3, 4], and malaria transmission in sub-Saharan Africa [5–7]. However, these strategies are only effective for adult vectors that rest indoors. The adult populations that rest or feed outdoors, and the immature stages that develop in water bodies, are not covered by the current vector control measures. More emphasis needs to be placed on controlling the aquatic stages, particularly as protection offered by current tools seems to be hampered by the emergence and spread of insecticide resistance across Africa [3, 8–12]. Larval source management (LSM) could provide an additional effective tool for the control of malaria vectors [13–16]. However, the use of LSM requires adequate knowledge of the larval ecology of the vectors, as well as characterization of their larval habitats in different ecological settings [17].

In Ghana*, Anopheles gambiae *sensu lato (s.l.), (incl. *An.gambiae *sensu stricto (s.s.)*, Anopheles arabiensis, Anopheles coluzzii*, and *Anopheles melas*), and *Anopheles funestus *s.s. are the major vectors of human malaria [18–20]. *Anopheles* mosquitoes breeds in varying habitats: *An. gambiae* breeds in clear, sunlit pools of water that can be natural or man-made, and permanent or temporary [21, 22], but, *An. funestus* prefer to breed in shady permanent or semi-permanent water bodies with floating or emergent vegetation. In order to make LSM feasible in Ghana, a better understanding of larval habitats and with their physicochemical parameters in endemic areas is crucial, especially as mosquito control is becoming increasingly difficult due to the spread of insecticide resistance [23, 24] and alteration in land use [25, 26].

Environmental alterations due to deforestation, vegetation clearance for agricultural activities, urbanization, and human population growth enhances the proliferation of larval habitats of malaria vectors [21, 27, 28]. In addition, created dams and irrigation systems in farming communities contribute immensely to the number of suitable larval habitats [29]. Changes in land use can *increase exposure* to *sunlight,* which in turn contributes to *the* availability of larval habitats with enabling conditions for mosquito larvae productivity [30–32]. However, the productivity and stability of larval habitats can be influenced by a myriad of factors: climatic (e.g., temperature and rainfall), environmental (e.g., vegetation cover, presence of predators and competitors, habitat size, and amount of sunlight) and the physicochemical properties of the water in the habitats [22, 29, 33].

The abundance of rainfall and vegetation cover in a breeding habitat can influence the distribution and density of larvae, which in turn can influence the abundance of adult *Anopheles* vectors [32, 34, 35]. Water temperature can also affect the development of eggs or allow the development of more microorganisms that are required by the larvae for food [32, 36]. Varying physicochemical properties of mosquito larval habitats can also have a direct and indirect effect on the biology, including oviposition, survival and spatial distribution of malaria vectors [37, 38].

Monitoring larval population dynamics in different land use settings over a period of time, and evaluating the ecology of larval mosquitoes has implications for vector control [13, 39, 40]. There is the need to understand the productivity and stability of larval habitats in order to model and predict if LSM is feasible. This study aimed to determine the stability and productivity of anopheline larval habitats in Dodowa (savannah-forest transition area) and Anyakpor (the coastal savannah area) in southern Ghana. The effect of environmental, physicochemical parameters, rainfall intensity on *Anopheles* larvae productivity and habitat stability was also studied in these two areas. The results will provide valuable information to help model and predict the suitability of larval control strategies in these malaria-endemic areas.

## Methods

### Study sites

**Fig. 1:**
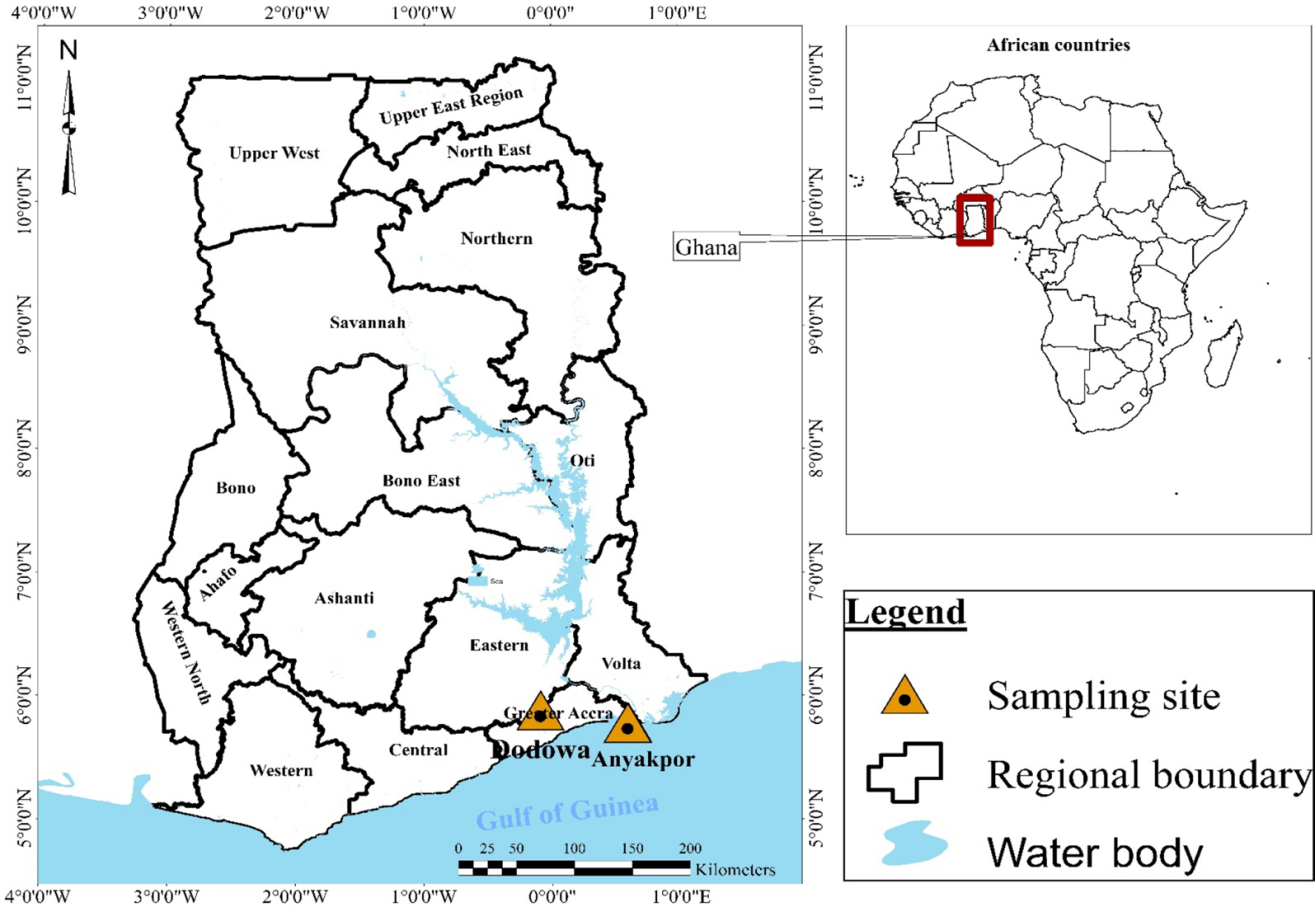
The study sites in southern Ghana

This study was undertaken in two rural areas: Anyakpor and Dodowa both located in the Greater Accra region of southern Ghana (Fig. 1). Ongoing studies in these sites have revealed diverse vector species composition and larval habitat types [41]. Malaria vectors reported in these sites include *An. gambiae* sensu lato (incl. *An. gambiae *s.s.*, An. arabiensis, An. coluzzii* and *An. melas*), *Anopheles pharoensis* and *An. funestus *s.s. as dominant in both sites [42].

Anyakpor (5°45′59.99ʺ N 0°36′59.99ʺ E) is a coastal village in Ada Foah in southern Ghana, and it is about 110 km from the city of Accra. It has a dry equatorial climate with temperatures ranging from 23 to 28 °C throughout the year and maximum temperatures reaching 33 °C. Its rainfall pattern is bimodal, with a long rainy season from April to June and a short rainy season from October to November with an annual rainfall of 750 mm. Anyakpor has coastal savannah type vegetation. Main farming activity in this area includes irrigated vegetable farming in low-lying areas consisting of dug-out wells and furrows that connect the wells. The area has a high-water table, and as a result water seeps into these dug-out wells, which creates breeding sites for mosquitoes.

Dodowa (5° 52′ 58.3212ʺ N 0° 5′ 52.9548ʺ W) is a community located in the savannah-forest transition zone in the Shai Osudoku District and is about 39 km from the city of Accra. It has an average temperature of 27 ℃ with a bimodal rainfall pattern like Anyakpor. It has secondary forest type vegetation with little original virgin forest left as a result of deforestation. Due to intense human activities in Dodowa, water accumulates at construction sites, unpaved roads and low-lying areas creating suitable larval habitats for mosquitoes.

### Measurement of habitat productivity and stability

**Fig. 2:**
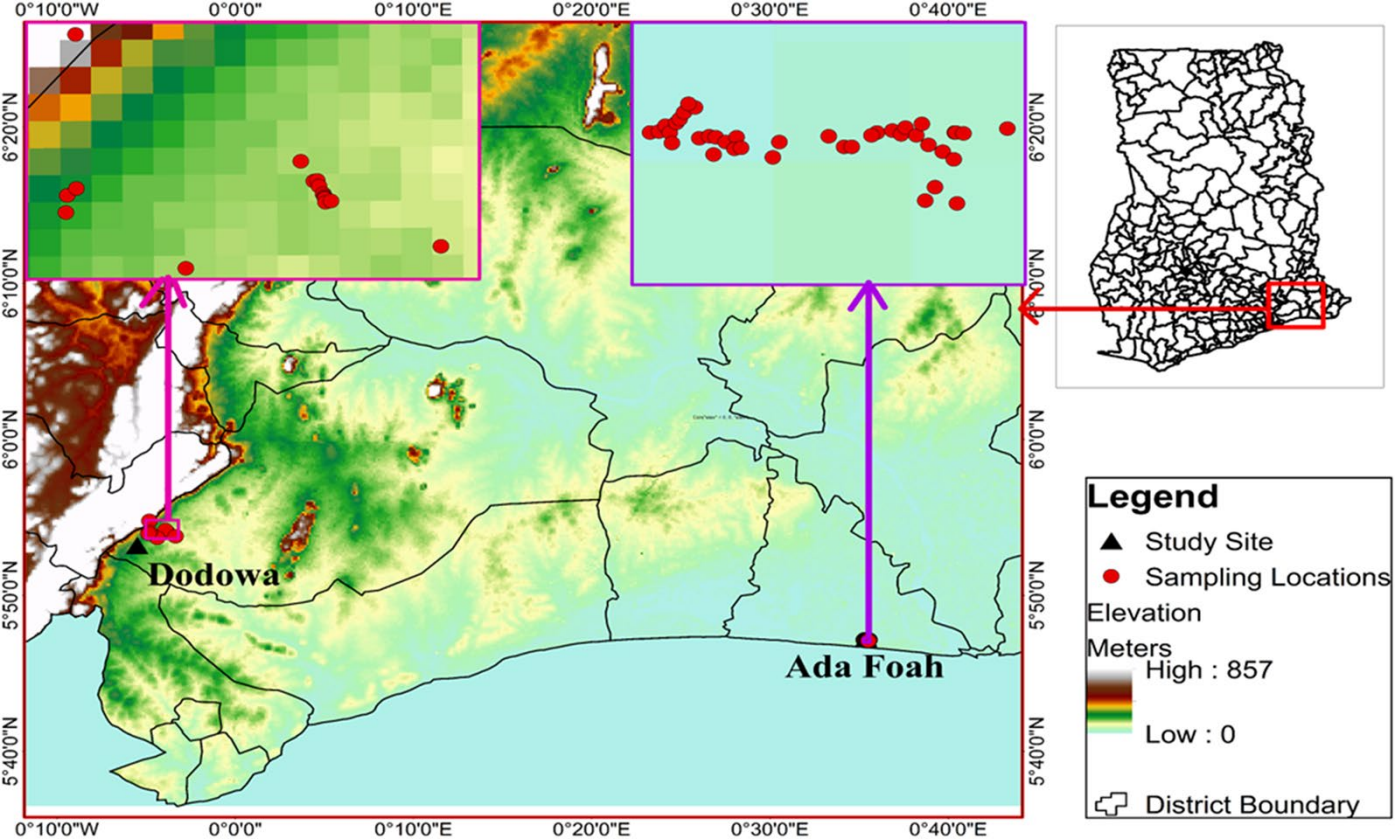
The study sites and locations of larval habitats

In September 2020, a preliminary survey was conducted in the study sites, and most of the larval habitats were found to be located approximately 2 km from the centre of each community (Fig. 2). These areas were selected for detailed study. Thorough searches of *Anopheles* larval habitats (e.g., man-made ponds, wells, swamps, furrows, puddles) were conducted and their locations mapped using a global positioning system (GPS: Garmin etrex^®^ 10) unit. A total of 59 larval habitats within the study sites, identified to consistently have immature mosquitoes, were chosen: 18 in Dodowa, and 42 in Anyakpor (Fig. 2). These 59 positive habitats were sampled for mosquito larvae once every two weeks from October 2020 to May 2021.

Individual habitats were numbered, and their surface area recorded along with the land use type and vegetation cover. The percentage of vegetation covering the surface of the habitats was visually estimated, and categorized as: 0 if vegetation was not present in the habitat, ≤ 24% surface coverage, and 25–49, 50–74 and 75–100% surface coverage [41]. Habitats were classified into land use types based on the activities taking place on the land where the larval habitat was found. During each survey, the physiochemical properties (temperature, pH, conductivity, dissolved oxygen, temperature, salinity, total dissolved solids (TDS) of the water in the habitats were measured on site using handheld multi-parameter tester (APERA Instrument PC60 Premium Multi-Parameter) based on guidelines provided by the manufacturer. The multi-parameter was calibrated and rinsed with distilled water before each use.

The larval habitats were grouped into temporary or permanent habitats. Temporary habitats were mainly rain-dependent and dried up when rain ceased for a while [43]. The permanent habitats was defined as habitats in which *Anopheles* larvae were found at least once, and contained water that was fed by natural underground sources throughout the sampling period [43].

Habitat stability was indicated by the availability of water in a habitat for 14 days, following previous reported studies that showed that egg-adult cycle of *An. gambiae *s.l. can be completed in this length of time [17, 37]. To determine productivity, the habitats were visited and examined once every two weeks for the presence of aquatic stages of anopheline and culicine mosquitoes. In addition, the area (length and width) of the water surface was measured and recorded in metres with a metal ruler and grouped as small (≤ 10 m^2^) or large (10–100 m^2^).

Mosquito larval surveys were also carried out to generate stage-specific estimates of larval densities. Water was dipped up to 20 times using a standard dipper (350 mL, BioQuip Products, Inc., CA, USA). When a habitat was too small to make 20 dips, water was dipped as many times as possible. Larval abundance was calculated as the number of larvae per number of dips made in each habitat. The number of larvae and pupae in each habitat was collected and recorded, with larvae classified as early instars (L1 and L2) or late instars (L3 and L4). Larval samples collected from each habitat were pooled into sterile plastic containers and transported to the insectary of the Department of Medical Microbiology, University of Ghana Medical School, where they were bred into adults. At the insectary, the larvae were fed on Tetramin^®^ fish meal and maintained at 27 ± 2 °C.

### Mosquito species identification

Emerged adult mosquitoes were morphologically identified using the taxonomic keys of Gillies and Coetzee [44]. *Anopheles gambiae* s.l. was further identified to sibling species and molecular forms using polymerase chain reaction (PCR) [45, 46].

### Meteorological data

For the entire study period, precipitation data were obtained from Ghana Meteorological Service (https://www.meteo.gov.gh/). The weather station located in Anyakpor and Akropong measured the regional daily precipitation throughout the study period.

### Data analysis

Data were entered in Microsoft Excel and analysed using STATA v15 (StataCorp. 2017). Descriptive statistical analysis was done to compare the presence of the various habitat types and larval densities in the different study sites and seasons. Larval densities were calculated by dividing the total number of larvae collected by the total number of dips taken.

The density of *Anopheles* mosquito larvae was compared among the various larval habitats and study sites. The Mann–Whitney U and the Kruskal–Wallis test were used to test the associations between larval densities in the two different sites. The Chi-square and Fisher's exact tests were first used to test the association between two categorical variables. Multiple logistic regression was then performed to assess the association between the habitat characteristics with categorical data and the presence/absence of *Anopheles* larvae. To find out whether rainfall intensity had any impact on the presence of *Anopheles* larval abundance, the average bi-weekly rainfall (mm) was calculated and then compared to the mean larval density using linear regression in both study sites, and Spearman’s correlation was used to determine the correlation between occurrences of mosquito larvae.

## Results

### Habitat characteristics and presence of mosquito larvae

Anyakpor (the coastal savannah zone) had more larval habitats (69.49%; 41/59) than Dodowa (30.5%; 18/59) in the savannah-forest transition zone. In the total number of times the larval habitats were sampled, 38.6% (168/435) of the habitats were inhabited by *Anopheles* larvae, while 61.38% (267/435) were not (Table 1). *Anopheles* larvae were mostly present in puddles (76.7%; 33/43) and furrows (58.3%; 7/12), followed by wells 40% (78/193) and man-made ponds 21.38% (31/145) (Table 1).

**Table 1:** Habitat characteristics and the occurrence of *Anopheles* larvae from October 2020 to May 2021 in two sites in southern Ghana

Characteristics	Categories	*Anopheles* present no/Total No. (%)	*p*-value
Study site	Anyakpor	112/343 (32.7)	*p* = 0.000 χ^2^ = 22.3392
	Dodowa	56/92(60.8)	
Habitat type	Man-made pond	31/145 (21.4)	*p* = 0.000 χ^2^ = 36.5267
	Well	87/214 (40.4)	
	Swamp	10/21 (47.6)	
	Puddle	33/43 (76.7)	
	Furrow	7/12 (58.3)	
	Total	168/435 (38.6)	
Land-use type	Farmland	119/363 (32.8)	*p* = 0.000 χ^2^ = 24.3645
	Road	24/40 (60.0)	
	Compound/Home	9/9 (100)	
	Water body	16/23 (66.7)	
Presence of culicines	Absent	112/308 (36.4)	*p* = 0.132 χ^2^ = 2.2671
	Present	196/308 (63.6)	
Habitat size categorized	< 10 m	133/323 (41.2)	*p* = 0.033 χ^2^ = 6.8114
	10–100 m	31/106 (29.3)	
	> 100 m	4/6 (66.7)	
Vegetation cover categorized	None	54/123 (43.9)	*p* = 0.001 χ^2^ = 19.9549
	< 24%	44/83 (53.0)	
	25–49%	19/46 (41.3)	
	50–74%	21/59 (35.6)	
	75–100%	30/124 (23.2)	
Presence of algae	Absent	106/284 (37.3)	*p* = 0.446 χ^2^ = 0.5804
	Present	178/284 (62.7)	
Surface debris	Absent	43/126 (34.1)	*p* = 0.219 χ^2^ = 1.5110
	Present	83/126 (65.9)	
Emergent plant	Absent	107/252 (42.5)	*p* = 0.014
	Present	145/252 (57.5)	χ^2^ = 3.7254

Five different habitat types (man-made pond, well, swamp, furrow, puddle) were encountered and recorded during the study. The likelihood of finding *Anopheles* larvae was higher in wells (OR = 2.085 (1.042–4.173); *p* = 0.038) than in man-made ponds (Table 2). Habitats with no vegetation had on average twice as many *Anopheles* larvae (B = 2.284 (0.792–3.779) than habitats with some vegetation cover (1 to 24%) (Additional file 1: Table S1). *Anopheles* larvae were more likely to be encountered in habitats which were 151–200 m from nearest human settlement (OR = 0.345; 95% CI 0.141–0.844: *p* = 0.020), compared to habitats that were more than 200 m from human settlement (Table 2). However, *Anopheles* larval density increased by a small margin for every metre a habitat was from human settlement (B = 0.023 (0.002–0.043); *p* < 0.05) (Additional file 1: Table S1). The presence of algae and land use type had no significant effect on the presence of *Anopheles* larvae).

**Table 2:** Logistic regression of larval habitat characteristics and the presence of *Anopheles* larvae from October 2020 to May 2021 in two sites in southern Ghana

*Anopheles* presence	aOR	St. Err	t-value	p-value	[95% Conf. Interval]	Sig.
Habitat type						
Man-made pond						
Well	2.085	0.738	2.08	0.038	1.042–4.173	^**^
Swamp	2.017	1.225	1.16	0.248	0.614–6.631	
Puddle	2.248	1.500	1.21	0.225	0.608–8.311	
Furrow	1.212	0.926	0.25	0.801	0.271–5.420	
Site						
Dodowa	1.000					
Anyakpor	0.561	0.424	− 0.77	0.444	0.128–2.463	^**^
Land-use type						
Farmland	1.000					
Road	1.031	0.818	0.04	0.969	0.218–4.884	
Compound/home	1.000					
Water body	3.414	2.957	1.42	0.156	0.625–18.641	
Habitat size						
< 10 m	1.000					
10–100 m	0.492	0.201	− 1.73	0.083	0.220–1.097	^*^
> 100 m	0.973	1.052	− 0.03	0.980	0.117–8.096	
Vegetation cover						
None	1.000					
< 24%	1.331	0.412	0.93	0.355	0.726–2.440	
25–49	0.780	0.328	− 0.59	0.554	0.342–1.778	
50–74	0.554	0.222	− 1.47	0.141	0.253–1.215	
75–100	0.208	0.077	− 4.24	0.001	0.101–0.430	^***^
Culex presence						
Absent	1.000					
Present	1.669	0.480	1.78	0.075	0.950–2.933	^*^
Algae						
Absent	1.000					
Present	1.202	0.385	0.57	0.565	0.642–2.253	
Surface debris						
Absent	1.000					
Present	0.609	0.174	− 1.73	0.083	0.348–1.068	^*^
Emergent plants						
Absent	1.000					
Present	0.885	0.282	− 0.38	0.702	0.474–1.652	
Distance to nearest settlement (m)						
201–250	1.000					
151–200	0.345	0.158	− 2.33	0.020	0.141–0.844	^**^
101–150	0.779	0.400	− 0.49	0.626	0.285–2.129	
51–100	1.762	2.311	0.43	0.666	0.135–23.039	
0–50	1.000					

*aOR* adjusted odds ratio

^***^p < *0.01*

^**^p < *0.05*

^*^p < *0.1*

### Mosquito vector larval presence and species composition at the study sites

A total of 13,681 mosquito larvae belonging to two genera (*Anopheles* and *Culex* species) were collected from 59 different larval habitats in Anyakpor (the coastal savannah zone) and Dodowa (savannah-forest transition zone) over the 30 weeks. Among the total mosquito larvae collected from the larval habitats in Anyakpor and Dodowa, 22.6% (3095/13,681) were anophelines and 77.4% (10,586/13,681) were culicines (Table 3). In Anyakpor, of a total 11,403 mosquito larvae collected from the different habitats, 77.8% (8869/11,403; 95% CI 77.0–78.5) were *Culex* and 22.2% (2534/11,403, 95% CI 21.5–23.0) were *Anopheles* larvae (Table 3). Of a total 2,278 mosquito larvae collected from the different habitats in Dodowa, 75.4% (1717/2278, 95% CI 73.5–77.12) were *Culex* and 23.9% (545/2278, 95% CI 21.5–23.0) were *Anopheles*. Of the 3,095 *Anopheles* mosquitoes collected overall, 99.5% (3079/3095) were *An*. *gambiae *s.l., 0.5% (14/3095) were *Anopheles rufipes* and 0.1% (2/3095) were *An*. *pharoensis*. A sub-sample of 559 *An. gambiae *s.l. from the two study sites were analysed for the identification of their respective sibling species. Overall, *An. coluzzii* accounted for 71% (95% CI 67.4–75.1), followed by *An. gambiae s.s.* [23% (95% CI 19.2–26.3)], and *An. melas* [6% (95% CI 3.9–7.9)]. In Anyakpor, 30% (387/2534) of *An. gambiae *s.l. were discriminated into sibling species and of these, 70.7% (284/387, 95% CI 68.6–77.7) were *An. coluzzii*, 19.9% (70/387, 95% CI 14.5–22.4) were *An. gambiae* and 8.8% (31/387, 95% CI 5.6–11.3) were *An. melas* (Table 3). *Anopheles melas* was only found in the coastal town of Anyakpor. In Dodowa, 30% (172/561) of *An. gambiae *s.l. analysed for their respective sibling species revealed 66.9% (115/172, 95% CI 59.23–73.7) were *An. coluzzii*, and 32.6% (56/172, 95% CI 25.7–40.2) were *An. gambiae*.

**Table 3:** Distribution of mosquito larvae at different study sites in southern Ghana from October 2020 to May 2021

Mosquito larvae	Number (%)		Total
	Anyakpor	Dodowa	
*An. gambiae *s.l	2,534	545	3079
*An. pharoensis*	0	2	2
*An. rufipes*	0	14	14
Culex	8,869	1717	10,586
Total	11,403	2278	13,681

*Anopheles gambiae *s.l	Anyakpor	Dodowa	Total (%)
*An. gambiae* s.s	70 (19.9%)	56 (32.6%)	126 (23)
*An. coluzzii*	284 (70.7%)	115 (66.9%)	399 (71)
*An. melas*	31 (8.8%)	0	31 (6)
Unidentified *An. gambiae* species	2 (0.6%)	1(0.5%)	3 (0.5)
Total	387	172	559

The mean larval density in Anyakpor was higher (4.779 larvae/dip, 95% CI 3.67–6.22) than in Dodowa (0.996 (95% CI 0.663–1.495) larvae/dip) (p = 0.0128, χ^2^ = 6.12, df = 1). The types of habitats in this study affected both the presence (p < 0.000, χ^2^ = 36.53, df = 4) of *Anopheles* mosquitoes. Overall, wells were the most productive habitats (with 6.44 (95% CI 5.0–8.31) larvae/dip), followed by furrows (4.18 (95% CI 2.75–6.36) larvae/dip) and man-made ponds (1.20 (95% CI 0.671–2.131) larvae/dip). In Anyakpor, furrows (7.16 (95% CI 3.96–12.93) larvae/dip) and wells (6.0 (CI 4.72–7.62) larvae/dip) were the most productive larval habitats. While in Dodowa, furrows (4.14 (95% CI 2.08–8.24) larvae/dip) and swamps (1.30 (95% CI 0.25–6.88) larvae/dip) were the most productive larval habitats.

### Effect of rainfall on habitat stability and *Anopheles* larval densities

**Fig. 3:**
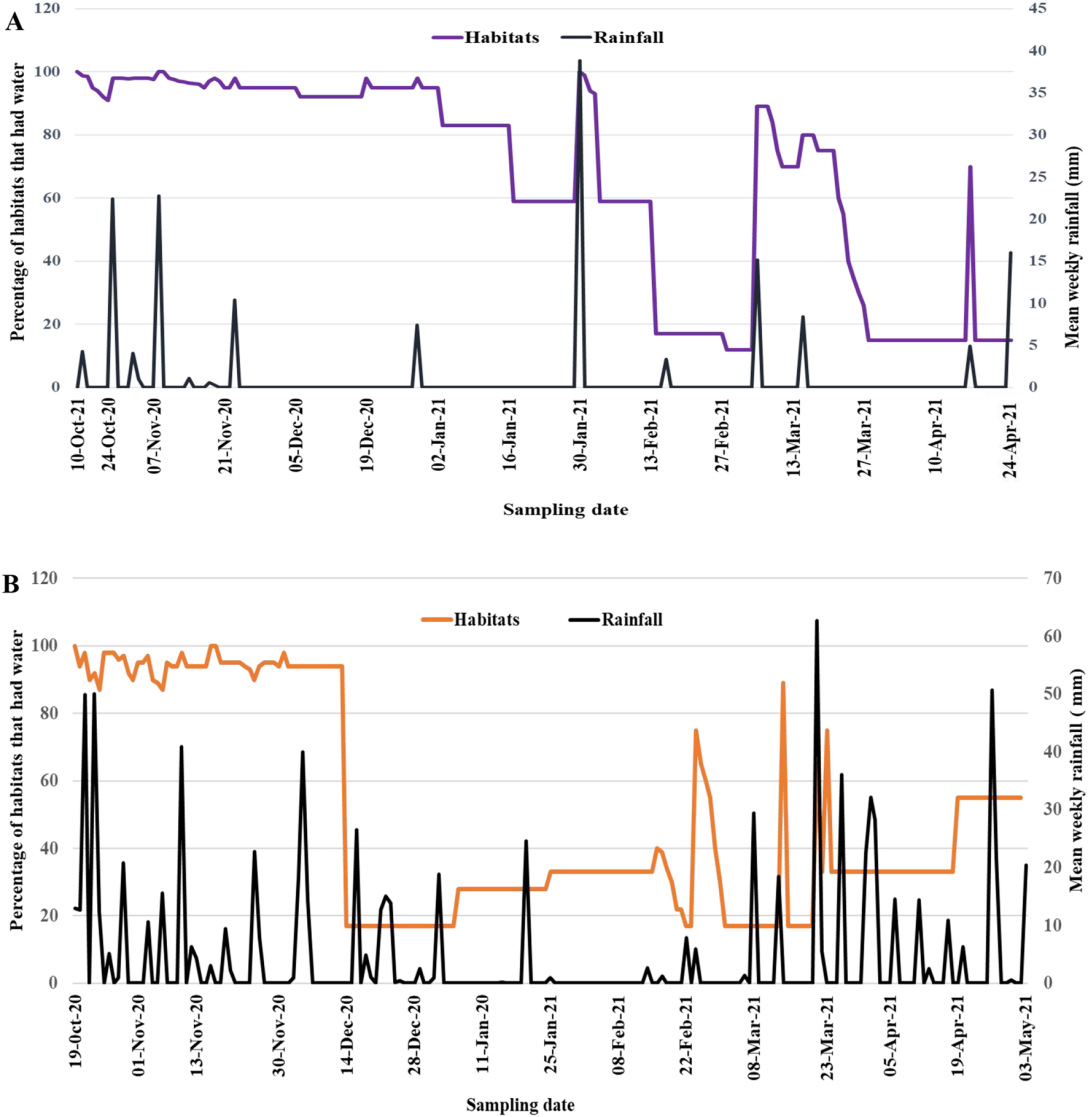
Temporal variations in mean cumulative rainfall (mm) and the percentage of habitats in **A** Anyakpor and **B** Dodowa that had water compared to the initial sampling period

Overall, rainfall influenced the availability of mosquito larval habitats at both sites (Fig. 3). However, the extent to which habitat frequencies fluctuated between rainy and dry season varied greatly between the two study sites. The mean length of weeks that a habitat contained water was significantly shorter in Dodowa site compared to Anyakpor (7.5 *vs* 10.7 weeks during rain and 2.9 *vs* 8.4 weeks dry season) (Table 4). The mean number of times that a habitat dried up in Dodowa was four times higher than that of the Anyakpor during the rainy season (4.0 *vs* 1.1), and two times more in the dry season (6.2 *vs* 3.9) (Table 4). Overall, the percentage of habitats that were aquatic on any given day in Dodowa and Anyakpor sites reduced during the rainy season from 95 *vs* 80.3% to 60.6 *vs* 55.0% in the dry season.

**Fig. 4:**
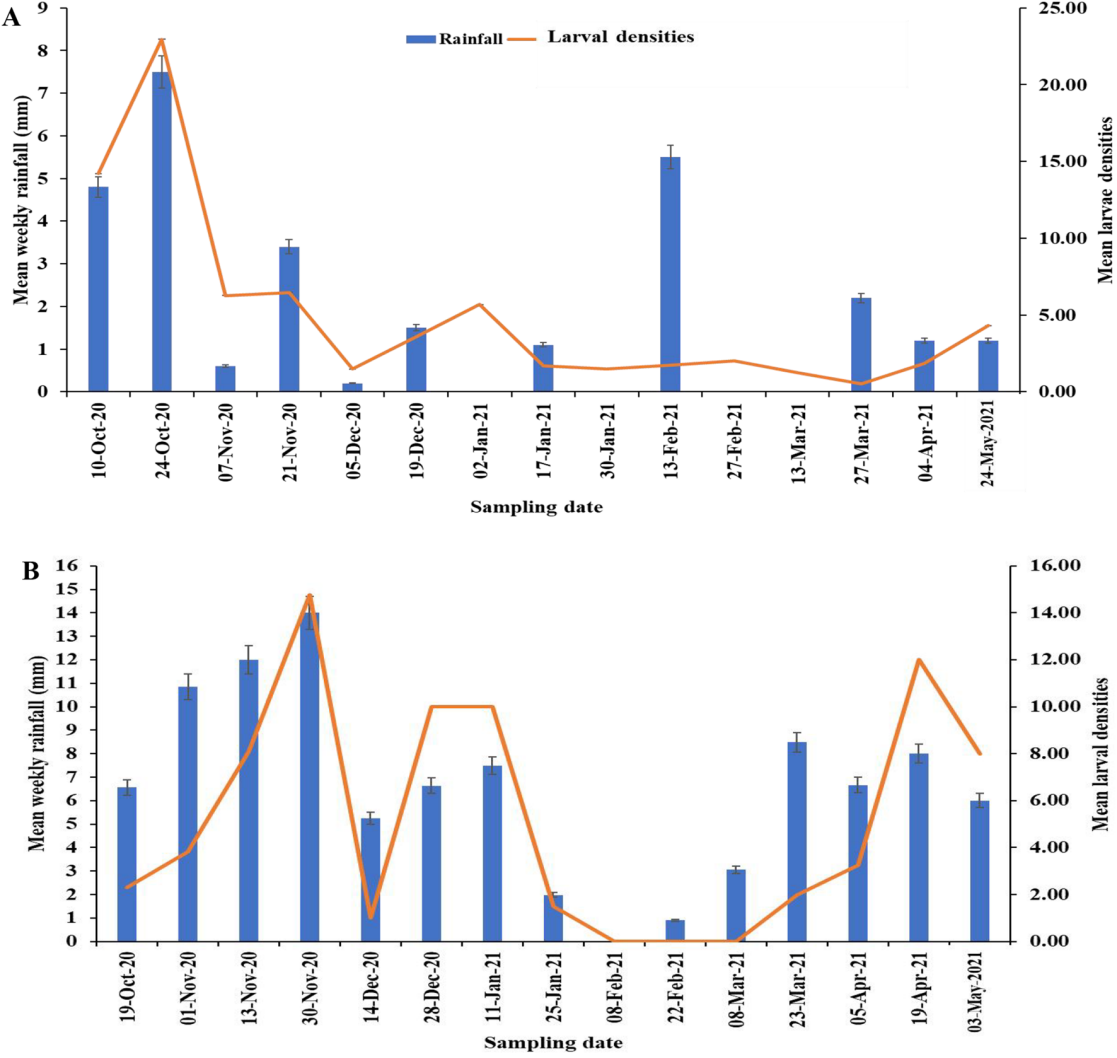
*Anopheles* larval densities and average weekly rainfall (mm) dynamics in **A** Anyakpor and **B** Dodowa throughout 30 weeks of field survey

**Table 4:** Multivariate MANOVA comparison of habitat stability between Dodowa and Anyakpor during rainy and dry season from October 2020 to May 2021

Parameters (mean ± SE)	Rainy season		Dry season	
	Dodowa	Anyakpor	Dodowa	Anyakpor
Mean duration that habitats remained aquatic	7.47 ± 2.5	10.68 ± 1.9	2.94 ± 3.5	8.439 ± 3.8
Mean number of times that habitats dried up	4.00 ± 1.7	1.14 ± 0.7	6.21 ± 1.7	3.92 ± 1.8
Mean daily percentage that habitats were aquatic	95.02 ± 24.6	80.30 ± 29.8	60.40 ± 24.6	55.0 ± 33.3

*Anopheles gambiae *s.l. larval densities exhibited temporal differences as shown in Fig. 4. Over all, high rainfall intensity led to an increase in mosquito larval densities (p = 0.001). The occurrence of *An. gambiae* larval densities was significantly associated (p < 0.05) with rainfall in Anyakpor (p = 0.0023) and in Dodowa (p = 0.047).

### The effect of physicochemical parameters on *Anopheles* larval presence

The results of analysis using Spearman’s *rho* coefficient analysis revealed the overall presence of *Anopheles* larvae was significantly influenced by elevated levels of pH (*rho* = 0.119). Further, increasing level of conductivity (*rho* = 0.246) and total dissolved solids (TDS) (*rho* = 0.227) had a positive and significant correlation with *Anopheles* larval density. In Anyakpor, *An. gambiae* larval density was significantly associated with conductivity (p = 0.011), TDS (p = 0.010), salinity (p = 0.0075), and pH (p = 0.0407). However, in Dodowa, there was no significant difference between all the studied physicochemical parameters and *An. gambiae* larval density (p < 0.05).

Table 5 shows the mean standard deviations of physicochemical parameters in the different categories of *Anopheles* larval habitats in Anyakpor and Dodowa. Compared to Dodowa, swamps and furrows in Anyakpor had the highest conductivity (10.4 ± 2.5 *vs* 11.25 ± 1.97 µs), TDS (7.23 ± 1.69 *vs* 7.43 ± 1.73 ppm), salinity (5.21 ± 1.26 *vs* 5.56 ± 2.01 ppm), and pH (7.05 ± 0.08 *vs* 7.02 ± 0.02).

**Table 5:** Distribution of mean levels of physicochemical parameters and *Anopheles gambiae* larval densities in Anyakpor and Dodowa from October 2020 to May 2021

Study site	Habitat type	No. of habitats	Temp. (°C)	pH	TDS (ppm)	Cond. (µs)	Salinity (ppm)
			Mean ± SD	Mean ± SD	Mean ± SD	Mean ± SD	Mean ± SD
Anyakpor	Swamps	3	33.44 ± 1.16	7.05 ± 0.08	7.23 ± 1.69	10.4 ± 2.50	5.21 ± 1.26
	Artificial ponds	12	32. 35 ± 1.13	7.10 ± 0.22	5.88 ± 2.05	8.62 ± 3.11	4.34 ± 1.67
	Wells	24	30. 58 ± 1.21	7.13 ± 0.11	2.75 ± 0.29	3.82 ± 0.51	1.89 ± 0.26
	Furrows	2	33.83 ± 0.67	7.02 ± 0.02	7.43 ± 1.73	11.25 ± 1.97	5.56 ± 2.01
Dodowa	Swamps	3	31.14 ± 1.62	7.68 ± 0.33	2.33 ± 0.85	3.23 ± 1.16	0.155 ± 0.05
	Artificial ponds	6	32.09 ± 2 .27	7.44 ± 0.24	3.55 ± 1.65	5.05 ± 2.38	0.24 ± 0.12
	Puddles	7	31.81 ± 1.83	7.60 ± 0.35	2.81 ± 1.67	3.71 ± 2.04	0.19 ± 0.01
	Furrows	2	31.20 ± 2.37	7.49 ± 0.05	1.87 ± 0.23	2.64 ± 0.32	0.13 ± 0.01

## Discussion

The availability of anopheline larval habitats and their productivity have important implications for malaria transmission [17, 21, 47, 48]. This study investigated the productivity, stability and effect of physicochemical parameters on larval density of malaria vectors in two sites in southern Ghana: Dodowa (savannah-forest transition area) and Anyakpor (the coastal savannah area). This study revealed *Anopheles* larval productivity and habitat stability was highly dependent on rainfall intensity. The results also indicated that presence of some physicochemical parameters in mosquito larval habitats at various levels increased mosquito vector larval density. This can contribute significantly to adult mosquito populations, vector distribution and disease transmission.

In this study, productivity of larval habitats was found to be positively correlated with rainfall at both sites, implying that when it rained, more habitats that are suitable for oviposition and larval development are produced. This can translate to a high production of adult mosquitoes and increase malaria transmission. Similar observations have been reported in Kenya by Imbahale et al. [48] and Sang et al. [49], where mosquitoes larvae productivity was found to be correlated with high rainfall intensity. However, studies by Munga et al. [50] and Afrane et al. [51] reported that higher amounts of rainfall correlated negatively with larval abundance and productivity.

For cost-effective mosquito larval habitat monitoring and control, understanding the impact of rainfall on the stability of larval habitats is of great importance [58, 59]. In this study, the stability of larval habitats was positively correlated with rainfall. The larval habitats in Anyakpor were more stable than the Dodowa site. The increase in stability of larval habitats in Anyakpor compared to Dodowa was because the water table is high in the former, and water seeps from the ground into the different habitat types allowing for larval development [52, 53]. Not only was stability of habitats lower in Dodowa, but also the frequency of positive anopheline larval habitats was significantly lower. Compared to Dodowa, Anyakpor is an intensive farming community, and as such, man-made ponds and wells created for irrigation purposes enhances the creation of stable aquatic habitats for the completion of the mosquito life cycle. Similar observations have been reported in Kenya by Minakawa et al. [54] and Himeidan et al. [17] where habitat stability was positively correlated with rainfall.

It was interesting to note that in the Anyakpor site, conductivity, TDS and salinity of the water in larval habitats had a significant influence on the larval density of *Anopheles* larvae compared to Dodowa site. High levels of conductivity and pH may be due to the application of agricultural fertilizers (organic or inorganic) and pesticides in Anyakpor [55–58]. The findings concur with previous studies conducted in northern Ethiopia [59] and Tanzania [37] which reported that high pH and conductivity were significantly associated with *Anopheles* larval density. However, this study’s findings are contradictory to studies conducted in Nigeria which reported that conductivity and TDS had no influence on *Anopheles* larval density [60], and low pH had a significant association with *Anopheles* density [61].

*Anopheles gambiae *s.l. was found to be more abundant than other *Anopheles* species (*An*. *rufipes* and *An*. *pharoensis*) in the two study sites. Sibling species of *An. gambiae *s.l. revealed a dominance of *An. coluzzii,* followed by few *An. gambiae *s.s. and *An. melas*. *Anopheles melas* was detected only in Anyakpor which has habitats that are fed by salty underground water from the sea, which *An. melas* prefers to breed in, unlike in Dodowa where habitats are fed by rainwater. A previous study conducted in southern Ghana has shown similar findings across similar habitat types [41, 62].

## Conclusion

The findings from this study reveal that the density of *Anopheles* larvae are affected by rainfall and physicochemical parameters present in their breeding sites. This calls for further studies to investigate the possible reasons for tolerance of high levels of physicochemical parameters among *Anopheles* mosquito populations. Additionally, this study revealed that human activity contributed to the majority of larval habitats at the study sites, and therefore, the involvement of the population would be highly beneficial in larval source management approaches.

## Supplementary Information

**Additional file 1: Table S1.** Generalized estimation equation regression of larval habitat characteristics and *Anopheles* larval densities from October 2020 to May 2021.

## Data Availability

The datasets used and/or analysed during the current study are available from the corresponding author on reasonable request.

## Abbreviations

CI: Confidence interval
GEE: Generalized estimating equation
OR: Odds ratio
SD: Standard deviation
Ppm: Parts per million
TDS: Total dissolved solids
µS: Microsiemens per centimetre
χ^2^: Chi square
Df: Degrees of freedom
