# Supplementary material for: Larval habitat stability and productivity in two sites in Southern Ghana
Source: Malar J. 2023 Mar 2;22:74. doi: 10.1186/s12936-023-04498-2 (PMC9983185; doi:10.1186/s12936-023-04498-2)
Supplement: Supplementary file 1 — Additional file 1: Table S1. Generalized estimation equation regression of larval habitat characteristics and Anopheles larval densities from October 2020 to May 2021. [file 12936_2023_4498_MOESM1_ESM.docx]

**Table 1 (S1) Generalized estimation equation regression of larval habitat characteristics and *Anopheles* larval densities from October 2020 to May 2021**

| ***Anopheles* Larval Density** | Adjusted B. | St. Err. | t-value | p-value | [95% Conf Interval] | | | Sig |
| --- | --- | --- | --- | --- | --- | --- | --- | --- |
| **Site** |  |  |  |  |  |  |  | |
| Dodowa | 1 |  |  |  |  |  |  | |
| Anyakpor | -4.218 | 2.099 | -2.01 | 0.044 | -8.331 | -0.104 | ** | |
| **Vegetation cover** |  |  |  |  |  |  |  | |
| None | 1 |  |  |  |  |  |  | |
| <24% | 2.284 | 0.761 | 3.00 | 0.003 | 0.792 | 3.776 | *** | |
| 25 – 49% | -0.339 | 0.974 | -0.35 | 0.727 | -2.248 | 1.569 |  | |
| 50 – 74% | -0.359 | 0.936 | -0.38 | 0.702 | -2.193 | 1.476 |  | |
| 75 – 100% | -0.654 | 0.781 | -0.84 | 0.402 | -2.184 | 0.876 |  | |
| **Habitat size** |  |  |  |  |  |  |  | |
| <10m | 1 |  |  |  |  |  |  | |
| 10 – 100m | -0.373 | 0.796 | -0.47 | 0.640 | -1.934 | 1.188 |  | |
| >100m |  | . | . | . | . | . |  | |
| **Habitat type** |  |  |  |  |  |  |  | |
| Man-made pond | 1 |  |  |  |  |  |  | |
| Well | 1.573 | 0.785 | 2.00 | 0.045 | 0.034 | 3.112 | ** | |
| Swamp | 0.108 | 1.344 | 0.08 | 0.936 | -2.527 | 2.742 |  | |
| Puddle | 0.497 | 1.324 | 0.38 | 0.708 | -2.098 | 3.092 |  | |
| Furrow | 1.292 | 1.612 | 0.80 | 0.423 | -1.869 | 4.452 |  | |
| **Presence of Culicines** |  |  |  |  |  |  |  | |
| Absent | 1 |  |  |  |  |  |  | |
| Present | 0.195 | 0.654 | 0.30 | 0.765 | -1.087 | 1.478 |  | |
| **Algae** |  |  |  |  |  |  |  | |
| Absent | 1 |  |  |  |  |  |  | |
| Present | -0.462 | 0.692 | -0.67 | 0.504 | -1.819 | 0.895 |  | |
| **Surface debris** |  |  |  |  |  |  |  | |
| Absent | 1 |  |  |  |  |  |  | |
| Present | -0.465 | 0.627 | -0.74 | 0.458 | -1.693 | 0.763 |  | |
| **Emergent plants** |  |  |  |  |  |  |  | |
| Absent | 1 |  |  |  |  |  |  | |
| Present | 0.152 | 0.693 | 0.22 | 0.827 | -1.206 | 1.510 |  | |
| **Distance to human settlement** | 0.023 | 0.010 | 2.19 | 0.028 | 0.002 | 0.043 | ** | |
| **Depth** | 0.006 | 0.005 | 1.11 | 0.266 | -0.004 | 0.016 |  | |
| **** p<0.01, ** p<0.05, * p<0.1* B= Regression coefficient | | | | | | |  |  |
